# Extracellular Vesicle Profiling Reveals Novel Autism Signatures in Patient-Derived Forebrain Organoids

**DOI:** 10.21203/rs.3.rs-6573757/v1

**Published:** 2025-05-13

**Authors:** Isidora Stankovic, Phillip Smit, Jonathan Cross, Paul Wolujewicz, David Greening, Dilek Colak

**Affiliations:** 1 Center for Neurogenetics, Feil Family Brain and Mind Research Institute, Weill Cornell Medicine, Cornell University, New York, NY, USA.; 2 Department of Medical Sciences, Frank H. Netter School of Medicine, Quinnipiac University, North Haven, CT, USA.; 3 Baker Heart and Diabetes Institute, Melbourne, VIC 3004, Australia.; 4 Department of Biomedical Sciences, School of Health Sciences, Quinnipiac University, Hamden, CT, USA.; 5 Baker Department of Cardiovascular Research, Translation and Implementation, La Trobe University, Bundoora, VIC, 3086, Australia.; 6 Baker Department of Cardiometabolic Health, University of Melbourne, Melbourne, Victoria, Australia.; 7 Center for Neurogenetics, Feil Family Brain and Mind Research Institute, Weill Cornell Medicine, Cornell University, New York, NY, USA.; 8 Gale and Ira Drukier Institute for Children’s Health, Weill Cornell Medicine, Cornell University, New York, NY, USA.

## Abstract

Autism Spectrum Disorder (ASD) affects 1 percent of the world’s population with an increased prevalence of 178 percent since 2000. Although altered synaptic function putatively accounts for many of the abnormalities seen in ASD, the specific molecular mechanisms underlying this disorder remain poorly defined. A growing body of evidence suggests that extracellular vesicles (EVs), specifically exosomes, play a critical role in cellular communication within the brain. While they have been implicated in various types of diseases from cancer to neurodegeneration, their involvement in ASD remains largely unexplored. In this study, we utilized patient-derived cortical organoid models to characterize EVs secreted by human three-dimensional (3D) tissue and defined their cargo. Our study reports, for the first time, alterations in ASD organoid-derived EVs in comparison to healthy control cortical EVs. By utilizing small RNA sequencing, proteomics, nanoparticle tracking and microscopy, we provide a comprehensive characterization of the cargo carried by EVs secreted from human 3D forebrain models. Our findings reveal substantial differences both in the RNA and protein content of ASD-derived EVs, providing insight into disease mechanisms as well as highlighting the potential of exosome-based diagnostics and therapies for ASD.

## Introduction

Autism Spectrum Disorder (ASD) is a developmental disability characterized by impaired social communication, restrictive repetitive behaviors, and quite often, cognitive deficits (1). ASD affects 1 in 36 children alone in the US, with a gender ratio of 4:1 affecting boys- a more than fourfold increase since 2000 (2). ASD is diagnosed using standardized assessment tools such as the Autism Diagnostic Observation Schedule (ADOS) in accordance with the criteria outlined in the Diagnostic and Statistical Manual of Mental Disorders (DSM-5) (1, 2). After half a century of studying ASD, we have yet to fully understand the initial insults, progression and pathophysiology of this disease, and therefore lack an effective treatment. Approximately 95% of these diagnoses are idiopathic, indicating the absence of a known genetic anomaly (3, 4). Genome-wide association and animal-model studies from syndromic ASDs suggest that dysregulated synaptic function accounts for most of the abnormalities found in ASD. A combination of factors - genetic abnormalities (2, 3), epigenetics (4, 5), immune system dysfunction (6, 7), maternal immune activation (8, 9, 10), and electrophysiological changes (11, 12) have been implicated in the etiology of ASD. Current treatments ranging from stem cell therapies to behavioral interventions, are only helpful in a portion of patients (2), and typically consist of a combination of behavioral therapies and interventions aimed to reduce symptoms that interfere with the quality of life (16). Given the brain’s cellular diversity and complexity, manipulating a single cell type is unlikely to yield effective therapeutic outcomes.

The majority of ASD cases exhibit polygenic susceptibility and involve coordinated cell communication amongst multiple cell types (17–20). Over the last decade, extracellular vesicles (EVs) have been identified as critical players in cell-to-cell communication (21) where they are produced by virtually all cell types and are released into the extracellular environment (21–23). To date, three main types of vesicles have been characterized: apoptotic bodies (500–2000 nm), microvesicles (50–1000 nm), and exosomes (40–200 nm). Although EVs differ in size, function, and biogenesis (21–23), all are secreted, carry molecules, and are involved in both short and long-distance cellular communication (24, 25). Additionally, EVs can cross the blood–brain barrier (BBB), and be detected peripherally, making them intriguing candidates in neurodevelopmental biomarker discovery (25), with recent evidence implicating a role for EVs during critical periods of brain development (20–25).

The smallest class of EVs are exosomes ranging from 40–200 nm in size and carry cargo composed of mainly proteins and micro-RNAs (21). Exosomes stimulate recipient cells through endocytosis and endosome fusion, direct release of cargo content, and signaling through cell surface receptors (23). Virtually all cell types in the brain release exosomes, including neurons, astrocytes, microglia, oligodendrocytes, and endothelial cells (25–32). Neurons and glia have been reported to release exosomes *in vitro* and *in vivo* (33–37). Trophic support of neurons from oligodendrocytes and astrocytes is thought to be conveyed by exosomes (35). Current evidence for exosome signaling in the brain points toward their role in transcriptional regulation (38), neurogenesis (39,40), synaptic plasticity (41,42), and neuroinflammation (43,44).

While studies found that cargo composition of exosomes is altered in diseases such as cancer and neurodegenerative disorders (22, 45, 46) exosome pathology in neurodevelopmental disorders is yet to be defined. A recent study examined exosomes from frozen postmortem prefrontal cortex and detected miRNA alterations in Schizophrenia and bipolar disorder (46). Exosomes have been shown to both carry hyperphosphorylated Tau between neurons and could seed amyloid-β aggregation in Alzheimer’s Disease (47, 48). *In vivo* studies demonstrated that the transmission of Tau via microglia-derived exosomes was more efficient than the transmission of the naked form of Tau (49). Recent studies have also used exosomal alpha-synuclein as a novel biomarker of early-onset Parkinson’s disease (50).

Although exosome research in neurodevelopmental disorders is still in its early stages, studies have implicated exosome dysfunction in ASD. In a 2D human cell culture model of Rett syndrome generated by MECP2-knockdown, it was shown that exosomes of MECP2-deficient cells lack proteins crucial for neuronal circuit development, while control exosomes were significantly enriched in proteins important for proliferation, neuronal development, and synaptic maturation (51). Treatment of MECP2-knockdown cultures with control exosomes rescued the defects in neuronal growth and differentiation (51). A similar study evaluated the efficacy of stem cell-derived exosomes on ameliorating autism-related behaviors in a BTB3 mouse model of idiopathic autism (52). Mice treated with exosomes either intranasally or intravenously showed increased social interactions and decreased repetitive behaviors 3 weeks after treatment. A later study administered stem cell-derived exosomes intranasally to a ShankB3 mouse model of autism and observed improvements in vocalization and repetitive behavior phenotypes, as well as increases in GABA receptor GABARB1 expression (53). While different studies highlight the ability of exosomes to improve autism-related behavioral phenotypes both in idiopathic and syndromic mouse models of ASD, the exact EV pathology and how it may contribute to disease initiation or progression are ill defined.

The emergence of induced pluripotent stem cell (iPSC) technology to derive monolayer brain cell types and 3D brain organoids from human donors offers a unique opportunity to study exosome pathology in ASD (54). Previously, iPSCs-derived cerebral organoids were used to study the neuropathologies of ASD and schizophrenia during early brain development (55–58). In the present study, we demonstrate successful isolation and characterization of EVs from ASD patient-derived forebrain organoids. We have identified significant alterations in the RNA and protein cargo of ASD EVs in comparison to healthy control EVs. Altered gene expression in EVs pointed towards disruptions in polyol pathways, protein synthesis, ubiquitination, and chromatin remodeling, all of which are linked to synaptic plasticity, cellular signaling, and protein homeostasis. Intriguingly, we identified several known ASD risk genes among the differentially regulated RNAs and proteins in EVs derived from ASD forebrain organoids. Our findings provide novel insights into the molecular mechanisms of ASD and supports the potential of EVs as therapeutic targets for the disorder.

## Results

### EVs secreted from CTRL and ASD organoids display similar density and morphology

**Figure 1.**
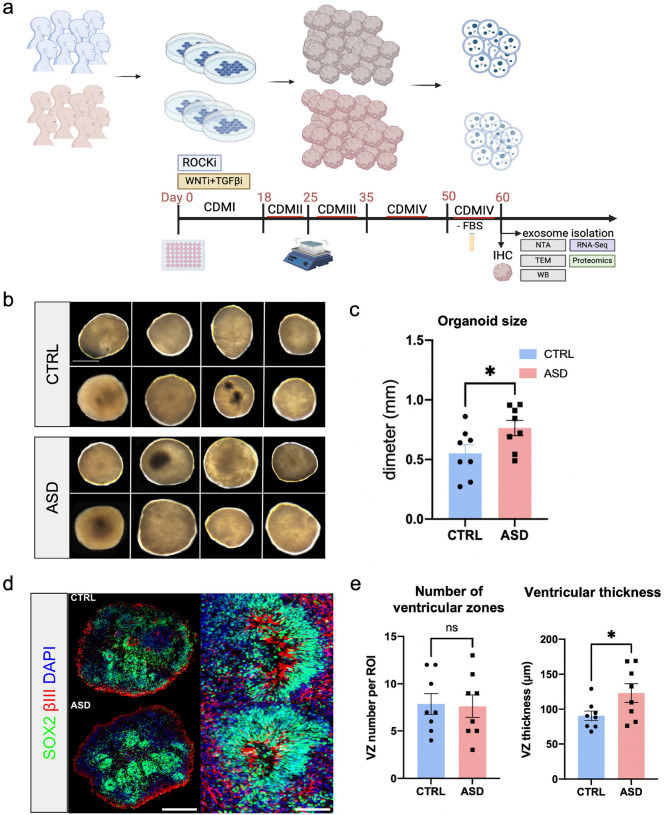
Forebrain organoids generated from iPSC lines derived from healthy controls and individuals with ASD **a)** Schematic depicting experimental design and timeline. Briefly, forebrain organoids were generated using a previously described protocol (59). At 60 DIV, media was collected, and EVs were isolated, followed by subsequent analysis. **b)** Light microscopy images of representative brain organoids at 60 DIV from all 16 lines used in the study. **c)** Bar graph depicts average size of organoids derived from CTRL and ASD lines (*n* = 8 lines per group; *n* = 3 organoids per line). **d)** Representative images of SOX2 (green), β-III-tubulin (red) and DAPI (blue) immunostaining in CTRL and ASD organoids to assess proliferative and neurogenic zones. **e)** Quantifications of ventricular-like zone density and thickness across 16 organoid lines used in the study (*n* = 8 lines per group; *n* = 3–10 organoids per line). Scale bar = 0.25 mm for b; 500 μm for d. Data is represented as mean ± SEM, *p* < 0.05.

To characterize EVs in early ASD brain development, we generated 3D dorsal forebrain organoids from human iPSCs. We sampled organoids from 16 unique human donors, comprising *n* = 8 healthy controls (CTRL) and *n* = 8 individuals with ASD (Figure 1a). Briefly, iPSCs from human donors were grown as monolayer cultures atop vitronectin-coated plates before being dissociated with Accutase to yield single-cell iPSC suspensions. Stem cell suspensions were correspondingly cultured into 3D embryoid bodies before being subjected to Cortical Differentiation Medium (CDM) and maintained as previously described (59). All 16 lines employed in the study formed organoids of similar morphologies (Figure 1b), but ASD-derived organoids were significantly larger in diameter than CTRLs (Figure 1b and 1c, **Figure S1a**). The ASD forebrain organoids exhibited similar phenotypes of increased progenitor pool and reduced neurogenesis as previously reported in this model system as well as in patients (Figure 1d and 1e, **Figure S1b-c**) (see Discussion).

**Figure 2.**
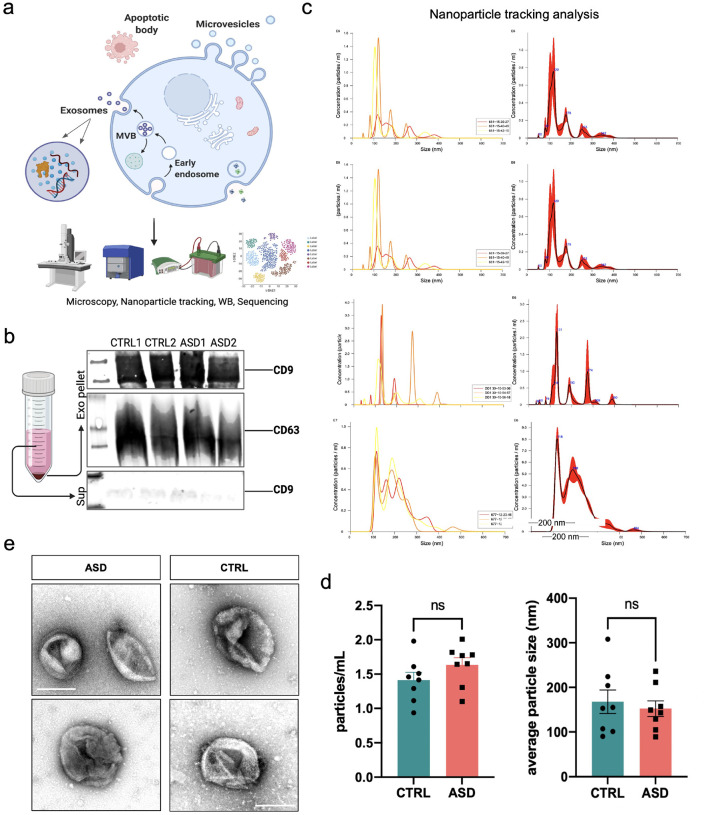
Exosome abundance in organoid-derived EVs **a)** Schematic of exosome validation methods. EVs were isolated from maintenance media of CTRL and ASD dorsal forebrain organoids at 60 DIV using a combination of Exo-isolation kit and centrifugation. EVs were then resuspended in either RIPA or PBS buffers, depending on downstream analysis. **b)** Representative Western blot examples in pellet and supernatant samples. Only pellets, not supernatant, were positive for exosome markers (CD9, CD63) indicating high efficiency in EV isolation. **c)** Representative graphs of nanoparticle tracking analysis (NTA). The x-axis represents concentration (particles/mL), and the y-axis represents particle size (nm). The top four are representative plots from CTRL lines while the bottom four plots are representative examples from ASD lines. The peaks typically appeared in the < 200nm range, representative of exosome size. **d)** Bar graphs depicting no significant difference in either average particle size or concentration between CTRL and ASD EVs at the NTA analysis. Each point on the graph represents an independent cell line. Data are represented as mean ± SEM. **e)** Representative TEM images of EVs isolated from CTRL and ASD organoid media. Vesicles typically displayed a canonical exosome cup/rose shape and size.

We next sought to determine whether EVs in ASD organoids are altered compared to EVs secreted by CTRL organoids. The schematic of experimental design and timeline is depicted in Figure 1a. To study ASD organoid-derived EVs, we generated CTRL and ASD organoids and collected the supernatant at day 60 of *in vitro* differentiation (60 DIV). Ten days prior to supernatant collection, FBS was removed from media to prevent contamination with FBS-derived vesicles (Figure 1a). EVs were isolated from culture media using a combination of commercially available kits (ThermoFisher) and ultracentrifugation. To validate EV purification, we followed the guidelines of the International Society for Extracellular Vesicles that recommends nanoparticle tracking analysis (NTA) for biophysical features, transmission electron microscopy (TEM) for high resolution imaging, and Western blot for validation of EV-specific proteins (60, 61) (Figure 2a). Western blot analysis verified the presence of exosomal markers CD9 and CD63 in the EV pellet post kit precipitation, but not in the processed supernatant sample, pointing to efficient EV isolation (Figure 2b). NTA revealed that across 16 lines, the majority of particles were <400nm (Figure 2c), with average particle sizes in the 90–250 nm range, representative of exosome size. NTA also allowed us to measure EV concentrations across samples. Intriguingly, ASD EVs and CTRL EVs did not differ in either size or density (Figure 2d). To visualize organoid-derived EVs, we employed TEM as previously described (61, 62). TEM images revealed vesicles exhibiting cup/rose-like morphologies, commonly associated with exosomes (Figure 2e). Taken together, we have used three techniques that independently assessed the size, morphology, or biochemistry of vesicles isolated from ASD and CTRL forebrain organoids. Although these analyses collectively suggest that the isolated EVs are mainly composed of exosomes, we cannot exclude the possibility that we have also isolated other types of EVs along with exosomes. Therefore, we simply refer to the purified vesicles as EVs.

### RNA and protein profiles are significantly different between ASD and CTRL EVs

**Figure 3.**
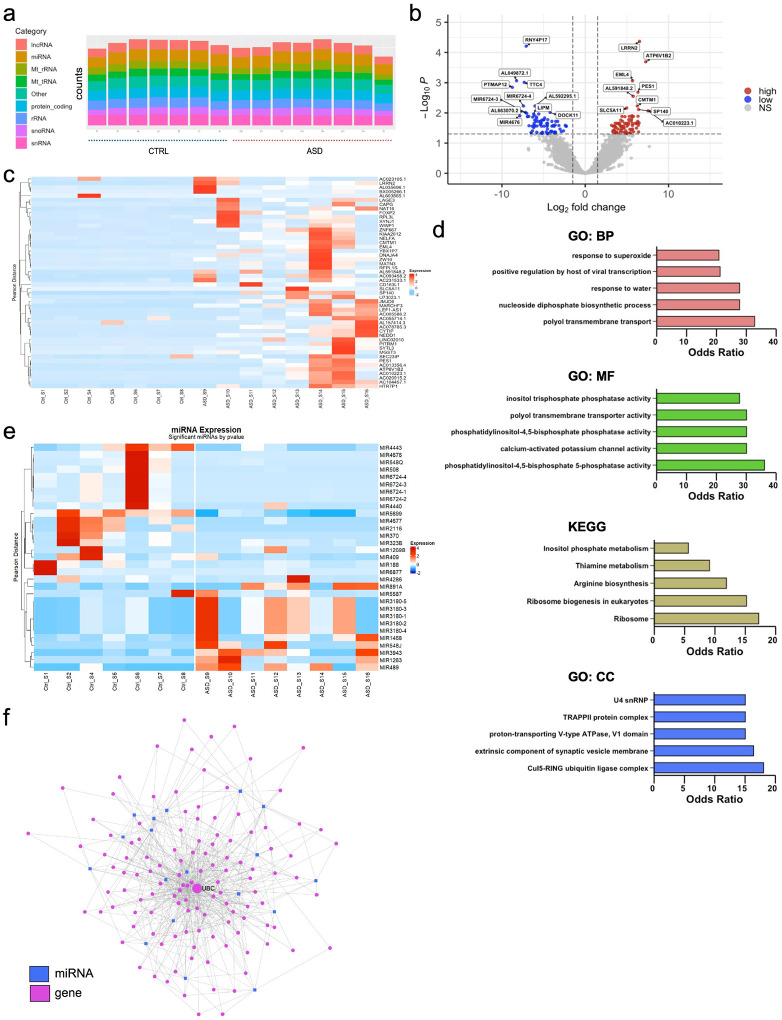
Differentially regulated RNAs point to translational control and ubiquitin activity in ASD EVs **a)** RNA counts by category per ASD and CTRL sample (n = 16 samples). **b)** Volcano plot displaying significantly expressed genes. Thresholds of significance used included Log2 FC > +/−1.5 and adjusted log10 Benjamini-Hochberg corrected *p* < +/− 0.05. **c)** Hierarchical and K-means heatmap of the top 50 differentially expressed RNAs in each sample (see supplementary materials for the full list of differentially regulated RNAs). **d)** Bar graph of GSEA enrichment terms from the respective pathway database. Terms ordered by descending odds ratio. Significant GSEA categories include i) Gene Ontology: Biological Processes (BP), ii) KEGG Kyoto Encyclopedia of Genes and Genomes, iii) Gene Ontology: Molecular Function (MF), iv) Gene Ontology: Cellular Component (CC). **e)** Hierarchical and K-means heatmap of the top 31 differentially expressed miRNAs in each sample; *p* < 0.05. **f)** miRNA–gene interaction network and hub gene analysis. Node size corresponds to the degree of connectivity, with larger nodes indicating higher numbers of interactions.

To compare the small RNA profiles of EVs derived from CTRL organoids and ASD organoids, we employed small RNA sequencing using the NEXTFLEX Small RNA Sequencing kit v4 which is designed to capture short RNAs (17–65 nucleotides) with 5´ monophosphorylated and 3´ hydroxylated ends. However, the kit also allows for the capture of larger molecules (<200 bp) by using the no-size selection protocol. We were able to detect RNAs from all 16 lines used in the study with dispersion estimate signifying low read count bias (**Figure S2a**). Of note, one line (CTRL line 3) showed low levels of RNA abundance (**Figure S2b**) and thus was excluded from downstream analysis. We first assessed RNA types present in the purified EVs and determined that organoid-derived EVs carry a large RNA heterogeneity. We incorporated the cut-off of 50 base pairs early in our pipeline and into the alignment steps. Intriguingly, in addition to non-coding RNAs, the analysis revealed that many of the fragments consistently aligned to protein coding RNAs as well as long RNAs both in CTRL and ASD EVs (Figure 3a). Given the heterogeneity of ASD, no RNAs were commonly altered across all ASD lines. However, several RNAs showed recurrent alterations across multiple ASD lines when compared to CTRL lines. We collectively identified 177 RNAs that were differentially regulated in ASD EVs compared to CTRL EVs. Of those 177 RNAs, 113 were protein-coding, with 58 upregulated and 55 downregulated. The remaining 64 differentially regulated RNAs were comprised of miRNAs, lincRNAs, snoRNAs, pseudogenes, and some uncharacterized transcripts (Figure 3b and 3c). The top 20 differentially regulated protein-coding RNAs in ASD are also shown (**Figure S2c**). To gain more insight into the function of differentially regulated RNAs, we grouped them based on their associated biological process, molecular functions, cellular components, and pathways (Figure 3d). Molecular function analysis predicted PIP2 phosphatase activity as the most differentially regulated molecular pathway. Among the 177 differentially regulated RNAs, ubiquitin ligase complex and synaptic vesicle membranes were the most enriched cellular components. In the differentially regulated non-coding RNA pool (64 RNAs), there were 31 miRNAs significant at the raw p-value level (Figure 3e). While some of these miRNAs were previously shown to be EV transported (63–65), most of the differentially regulated miRNAs were novel as EV cargo (**Figure S2d**). To predict the functional impact of varied miRNA profiles in EVs across all ASD lines, we performed a miRNA–target gene network topology analysis. The network revealed a highly interconnected regulatory architecture, with several hub genes targeted by multiple EV-enriched miRNAs (Figure 3f). Ubiquitin C (UBC) emerged as the most central node (degree = 99, betweenness = 4835.98), suggesting a pivotal role in EV-mediated signaling. Other top-ranking genes included heat shock proteins (HSP90AA1, HSP90AB1), transcription factors (TP53, HNF4A), and synaptic regulators (AKT1, CTNNB1). These hubs are implicated in stress response, proteostasis, chromatin remodeling, and synaptic plasticity—processes increasingly recognized as dysregulated in ASD. Our findings reveal that human forebrain organoid-derived EVs carry various RNA subtypes and that RNA content of ASD EVs is vastly different than CTRL EVs.

**Figure 4.**
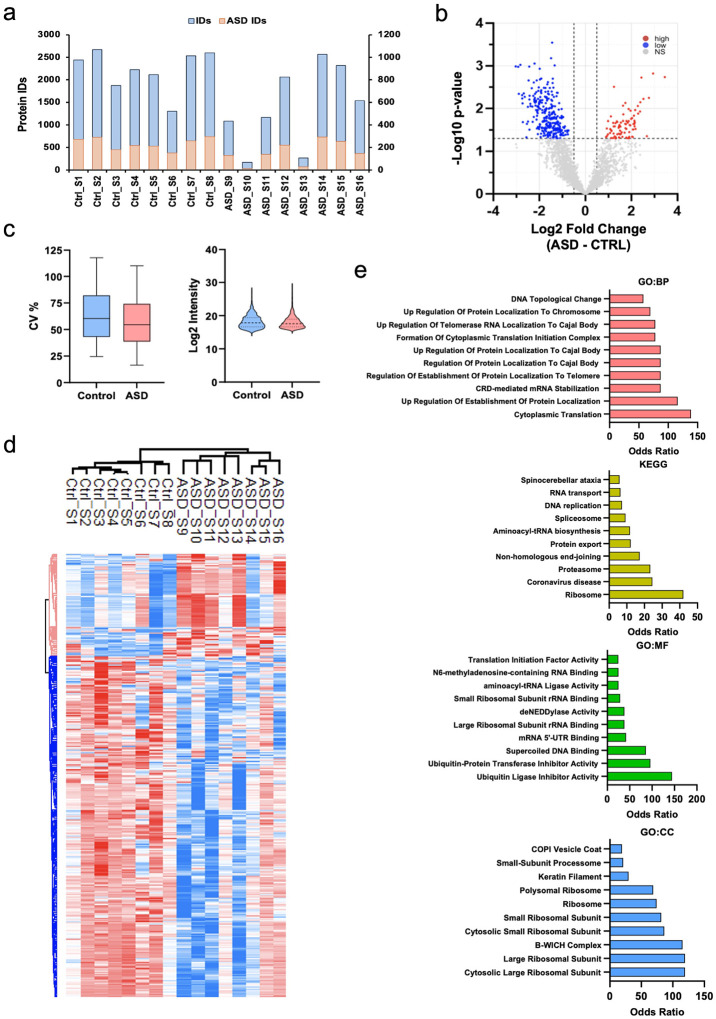
Altered protein expression comprises translation and protein degradation related categories in ASD EVs **a)** Protein counts by category per ASD and CTRL sample (n = 16 samples). **b)** Volcano plot displaying proteins expressed significantly. Thresholds of significance used included Log2 FC > +/− 1.5 and adjusted log10 Benjamini-Hochberg corrected *p* < 0.05. **c)** Coefficient of variance in CV and ASD lines, (n = 16 samples), non-significant. **d)** Clustered heatmap of 362 significant proteins based on a Student T-test analysis (*p* < 0.05). Values based on z-score normalization. **e)** Gene ontology analysis of significant proteins using enrichR. Top 10 categories selected (*p* < 0.05); odds ratio observed between ASD and CTRLs. Terms ordered by descending odds ratio. Significant GSEA categories include i) Gene Ontology: Biological Processes (BP), ii) KEGG Kyoto Encyclopedia of Genes and Genomes, iii) Gene Ontology: Molecular Function (MF), iv) Gene Ontology: Cellular Component (CC).

To compare protein profiles of EVs secreted from CTRL organoids and ASD organoids, we employed label-free mass spectrometry and successfully captured a large pool of proteins from all 16 lines (Figure 4a, 4b) with comparable variability (Figure 4c). Of note, two ASD lines showed lower protein capture than average. Similar to the RNA analysis, we did not find proteins that were commonly altered across all ASD samples. Collectively, 362 proteins were differentially regulated in ASD EVs in comparison to CTRL EVs with the majority exhibiting downregulation (279 downregulated and 83 upregulated) (Figure 4b, 4d). We grouped the differentially regulated proteins based on biological process, molecular function, cellular component, and pathway represented (Figure 4e). The gene ontology (GO) analysis predicted cytoplasmic translation and mRNA stabilization as the most altered biological processes. While molecular function analysis determined ubiquitin ligase and ubiquitin transferase inhibitor activity as the most differentially regulated molecular pathways, the top categories included ribosomal subunits and the B-WICH chromatin remodeling complex in cellular compartment analysis.

**Figure 5.**
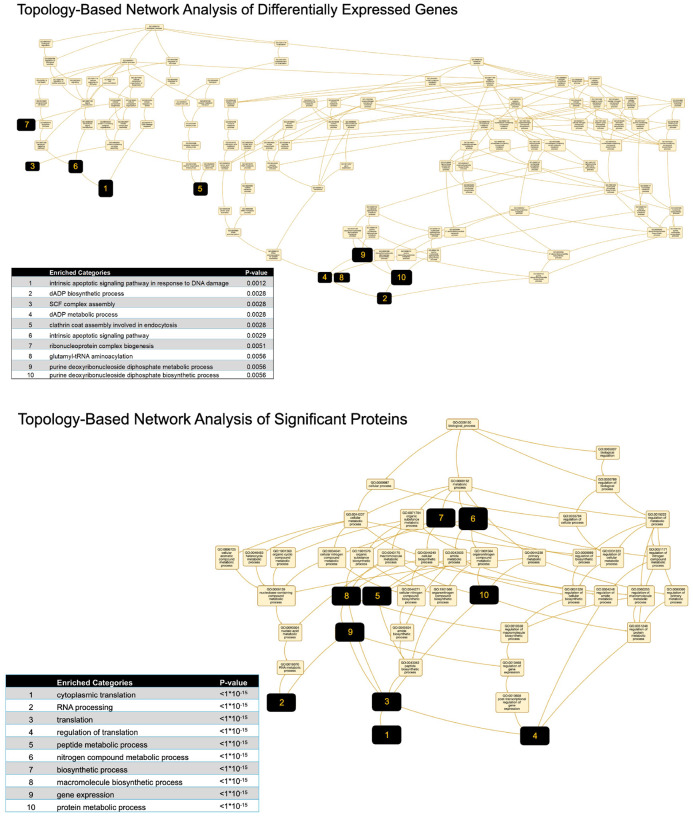
Altered pathways predicted by network analysis in ASD EVs Topology-based network enrichment analysis of differentially expressed genes and proteins in ASD-derived EVs. Top: Gene network analysis revealed 10 significantly enriched categories, including apoptosis, GDP metabolism, and clathrin-mediated endocytosis (*p* < 0.01). Bottom: Protein network analysis identified highly interconnected enrichment in translation, RNA processing, and biosynthetic pathways (*p* < 1×10−1⁵). Node size reflects the degree of connectivity within the GO network.

To determine if varied molecule expression in EVs across all ASD lines converge upon known biological pathways, we performed topology-based enrichment network analysis (Figure 5). For RNA cargo, enriched gene clusters were predominantly associated with apoptosis, metabolic regulation, and vesicular trafficking—highlighted by categories such as “intrinsic apoptotic signaling,” “GDP biosynthesis,” and “clathrin-coated endocytosis”. These findings suggest that ASD EVs may influence recipient cells by modulating survival pathways and intracellular transport. In parallel, analysis of differentially expressed proteins revealed a striking convergence on pathways related to translation and RNA processing. Highly enriched terms included “cytoplasmic translation”, “RNA processing”, “peptide metabolic process”, and “gene expression”, underscoring a coordinated disruption in protein synthesis machinery in ASD EVs.

### Differentially regulated molecules include ASD risk genes in ASD organoid-derived EVs

**Figure 6.**
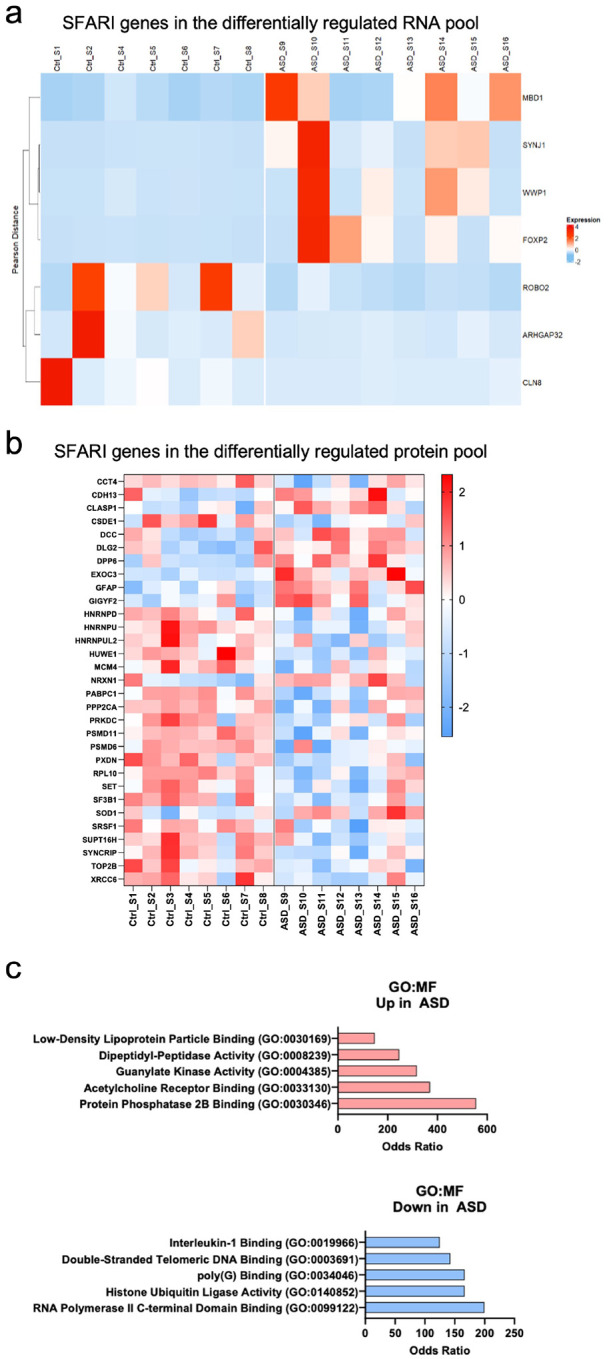
**Differentially regulated RNAs and proteins in ASD EVs include ASD risk genes a**) Heatmap of the significant SFARI genes among the differentially expressed mRNAs in each group. Values based on z-score normalization (*p* < 0.05). **b)** Heatmap of the significant SFARI genes among the differentially expressed proteins. Values based on z-score normalization (*p* < 0.05). **c)** enrichR gene ontology analysis of the significant SFARI risk genes among the differentially expressed proteins in ASD EVs. Top categories selected (*p* < 0.05); odds ratio observed between ASD and CTRL.

Lastly, we sought to determine whether differentially regulated molecules in ASD EVs include any known ASD risk genes. For this, we used the Simons Foundation Autism Research Initiative (SFARI) gene database, which focuses on genes implicated in ASD susceptibility. SFARI gene comparison pointed to 7 ASD risk genes in the differentially regulated RNA pool (Figure 6a). At the protein level, we found a significant abundance of differentially regulated SFARI risk genes in ASD EVs compared to CTRL EVs (Figure 6b). Notably, a majority of these proteins were downregulated in ASD and mostly involved in transcription, DNA damage, and chromatin structure (Figure 6c). In contrast, upregulated proteins were associated with neuronal excitability and calcium signaling. Collectively, these findings suggest that differential regulation of ASD risk genes in ASD-derived EVs reflects key disruptions in synaptic function, RNA processing, DNA stability, and synaptic integrity.

## Discussion

In ASD, neural circuits involved in social interaction, communication, and repetitive behaviors are disrupted, potentially due to imbalances in excitatory and inhibitory neurotransmission and altered connectivity. Development of the neuronal circuit and its function relies on a continuous crosstalk between neurons and non-neuronal cells. In the last decade, EVs have gained attention for their role in facilitating intercellular communication by delivering cargo such as RNA and proteins, which can influence a variety of cellular processes in recipient cells (66–71). The cargo within EVs is dynamic and can vary depending on the cell type of origin, suggesting that EVs may play a central role in modulating neurodevelopmental processes. While recent studies implicate EVs in neuronal phenotypes seen in ASD models with potential use of exosomes as a novel therapeutic tool, the pathology of EVs and its exact impact on the initial insults of the disorder are yet to be determined. Our study takes a critical step in understanding this dynamic by providing the first comprehensive characterization of EVs secreted by multicellular brain organoids derived from both ASD patients and neurotypical controls. By profiling the RNA and proteomic cargo of patient-derived EVs during neurodevelopment, we postulate how EV-mediated intercellular communication may be altered in ASD.

Adopting a protocol for the generation of human dorsal forebrain organoids (59), we generated hundreds of reproducible organoids across 16 different iPSC donors, 8 of which were derived from individuals with idiopathic ASD (Figure 1, see also Supplementary Table). The morphological phenotype in ASD-derived brain organoids, specifically the increased size, align with well-documented neurodevelopmental phenotypes observed both in *in vitro* organoid models of ASD and some patients (72, 73). This phenotype is consistent with the increased brain volume seen in a fraction of ASD patients, particularly during early childhood (74), supporting that brain organoid models could serve as a relevant representation of the abnormal developmental trajectories observed in ASD. The increased thickness of the ventricular zone in ASD organoids further supports this, as it aligns with similar findings reported by other groups in ASD organoids (75) and in patients, where abnormal changes in the subependymal zone have been linked to neurodevelopmental delays and irregular brain development (74). Despite the genetic variability in iPSC lines, our model system consistently recapitulates key aspects of early brain pathology in ASD. This reinforces the potential of brain organoids as a reliable platform for studying ASD and exploring therapeutic avenues for modulating early neurodevelopmental disruptions.

Here, we demonstrate the successful isolation of EVs from CTRL and ASD forebrain organoids using kit-based precipitation of EVs. Although recent studies have isolated EVs from CTRL organoids using varying protocols (76, 77) standardized methods for EV isolation remain elusive. Several isolation techniques are employed, such as ultracentrifugation and precipitation-based kits, each with its own advantages and applications (78–81). Given the limited culture size and heterogeneity of our iPSC lines, we opted for kit-based precipitation to maximize EV yield. We confirmed successful isolation of EVs by 3 different methods- NTA, TEM and Western blotting, ensuring consistent EV isolation across all lines. While we observed no major morphological differences in EVs between ASD and CTRL organoids, the overlap in size and surface markers between exosomes and other micro-vesicles made it challenging to distinguish specific EV subtypes with certainty. Thus, for the scope of this study, we focused on a deeper analysis of the RNA and proteomic cargo itself, rather than outer differences of EVs, aiming to uncover potential molecular differences in ASD.

In our analysis of the RNA cargo in EVs, we observed significant differences both in coding and non-coding RNA content between ASD and CTRL samples (Figure 3). Although most of the EV studies focus on miRNA content, EVs were also shown to carry mRNAs (82). In fact, we found that 58 coding RNAs were upregulated and 55 coding RNAs were downregulated in ASD EVs compared to CTRLs. These genes are grouped into clinically significant pathways such as polyols and phosphoinositide metabolism (Figure 3d). Recent findings have suggested that reducing polyol consumption can alleviate gastrointestinal and behavioral symptoms in ASD individuals (83) Similarly, phosphoinositides, particularly phosphatidylinositol 4,5-bisphosphate (PIP2) and its metabolites, play an important role in regulating cellular signaling pathways involved in neuronal development, synaptic plasticity, and neurotransmitter release (84, 85). Disruptions in phosphoinositide signaling could affect neuronal connectivity and synaptic function, both of which are central to the pathophysiology of ASD. In addition to coding genes, we also identified a range of upregulated noncoding RNA elements, particularly miRNAs, in ASD-derived EVs (Figure 3e, **Figure S2d**). Specifically, exosomal miRNAs were shown to play a pivotal role in regulating gene expression by modulating mRNA stability and translation (87, 88). For example, miRNA370, which was previously shown to increase permeability of the BBB (88) is altered in ASD EVs. Indeed, altered BBB permeability and associated inflammation have been observed in ASD (89). Our study also identified novel miRNAs in the context of EV cargo such as miRNA6724–1, miRNA6724–2, miRNA6724–3, and miRNA6724–4. Intriguingly, miRNA4286, which overall is upregulated in ASD EVs, has been linked to non-small cell lung cancer (90), and its overexpression was shown as an unfavorable prognostic marker in non-small cell lung cancer (91). Future directions may involve studies exploring the potential of miRNA expression in ASD EVs as diagnostic markers for this disorder, offering a non-invasive approach to early diagnosis and monitoring of neurodevelopmental conditions.

We also detected altered expressions of proteins involved in key cellular processes such as ubiquitination, translation, and ribosome biogenesis in ASD EVs (Figure 4). Perturbations in the ubiquitin-proteasome system, a critical pathway for protein turnover and the degradation of damaged or misfolded proteins, can lead to the accumulation of faulty proteins, triggering neuroinflammatory responses. This disruption is particularly relevant in the context of ASD, where chronic inflammation and immune dysregulation are increasingly recognized as contributing factors to neurodevelopmental impairments (92–94). Several lines of evidence suggest that alterations in the ubiquitin-proteasome system and other ubiquitin-related pathways may contribute to the onset or severity of ASD (95–100). Of note, proteins of the B-WICH complex, a regulator of chromatin remodeling (101–103), along with proteins involved in translation and ribosome regulation, compromised the top categories in GO analysis of altered EV proteome in ASD. In fact, whole exome sequencing studies employing large patient cohorts have revealed that the majority of ASD risk genes are chromatin and gene expression regulators (104). Similarly, altered translation has been implicated in various diverse types of ASD models (105–107).

Most importantly, our topology-based enrichment network analysis of varied molecule expression found in EVs across patient lines pointed to limited biological pathways most of which have been linked to ASD (Figure 5). For RNA cargo, enriched gene clusters were predominantly associated with apoptosis, metabolic regulation, and vesicular trafficking—highlighted by categories such as “intrinsic apoptotic signaling”, “GDP biosynthesis”, and “clathrin-coated endocytosis”. These findings suggest that ASD EVs may influence recipient cells by modulating cell survival pathways and intracellular transport. The network analysis of proteins with varied expressions across ASD EVs found enrichment in translational and gene expression regulation categories. This network analysis aligns with the findings from the GO analyses of differentially regulated EV proteins from individual lines, which collectively pointed to gene regulation pathways (Figure 4). Our findings are consistent with the hypothesis that dysregulation of translational control is a converging mechanism, not only in syndromic, but also in idiopathic forms of ASD (107).

Lastly, among the differentially regulated coding RNAs and proteins in ASD-derived EVs, several SFARI-based ASD risk genes were identified. Notably, the RNA of FOXP2, a transcription factor implicated in neurodevelopment and speech disorders (108), was differentially regulated- upregulated in some ASD EV lines and downregulated in others. Similarly, the RNA of the epigenetic regulator MBD1, whose deficiency is linked to autism-related behaviors in mice (109), was also among the altered ASD risk genes. MBD1 was upregulated in the majority of ASD lines compared to the majority of CTRL lines. In contrast, RNAs of certain ASD risk genes that were downregulated in ASD EVs are linked to axon guidance (i.e., ROBO2). Similarly, the CLASP1 protein, linked to cell polarization and axon guidance, was upregulated in EVs from the majority of ASD lines compared to CTRL EVs. However, most of differentially expressed EV proteins that are recognized as ASD risk genes were downregulated in ASD EVs. These proteins are primarily involved in RNA splicing, RNA processing, and protein synthesis. Together, these categories represented 75% of the downregulated ASD risk genes in the ASD EV proteome, further supporting the hypothesis that dysregulation of translational control is a converging mechanism in ASD.

## Conclusions

Our study, for the first time, reports a comparative analysis between EVs in human brain organoids derived from healthy controls and from individuals with ASD. EVs are recognized as regulators of brain development by facilitating cell-to-cell communication, and influencing processes like neurogenesis, synaptogenesis, and myelination, potentially playing a role in neurodevelopmental disorders. We identified significant alterations in RNA and protein content in ASD EVs at relatively earlier time points of brain organoid development. Gene ontology and network analysis pointed to RNA regulation, translation, DNA damage, and chromatin regulation, suggesting that EV pathology in ASD may contribute to impaired gene expression regulation, a hallmark of neurodevelopmental disorders. **While most of these processes have been linked to ASD pathology and etiology, our study identifies EV pathology and EV-mediated non-cell-autonomous regulation of brain development as a potential factor in ASD etiology.** Understanding non-cell-autonomous regulation of neuronal activity in ASD is crucial in addressing the underlying mechanisms of the disorder and for developing targeted therapies. Our findings not only advance our understanding of the role of EVs in ASD mechanisms but also sets the stage for exploring their potential as biomarkers, or therapeutic targets, in the treatment of ASD.

## Materials and Methods

### iPSC Lines

iPSC lines were purchased from NIH, CIRM repositories and Coriell Institute **(Supplementary Table 1).** Each repository characterized and validated cells as pluripotent and performed karyotyping to ensure the genomic integrity of each reprogrammed line. ASD lines MH0148698 and MH0148713 from a NIMH collection were genetically characterized and published (110). A total of 16 different iPSC lines (8 CTRL, 8 ASD) were utilized. All ASD samples were derived from non-syndromic cases and were not associated with any known genetic anomaly (see also Supplementary Table). All ASD iPSC lines were derived from males. To maintain consistency and based on availability, we also used CTRL lines that were derived from males except two lines. All iPSC lines were maintained on Vitronectin-coated plates and fed with Essential 8 (E8) + E8 supplement (ThermoFisher, CAT#: A1517001). All iPSC lines were cultured simultaneously to control for idiosyncratic culturing conditions. In all experiments, low passages (less than 18) were used, and all differentiations were derived from a single clone for each line.

### Dorsal forebrain organoid generation

Cerebral organoids were generated using a modified version of a previously published protocol (59). Briefly, on day 0, cultured human iPSCs, 80–90% confluent, were dissociated to single cells with Accutase (Gibco), and 9,000 cells per well were reaggregated in ultra-low cell-adhesion 96-well plates in Cortical Differentiation Medium (CDM) I, containing Glasgow-MEM (Gibco), 20% Knockout Serum Replacement (Gibco), 0.1 mM Minimum Essential Medium non-essential amino acids (MEM-NEAA) (Gibco), 1 mM pyruvate (Gibco), 0.1 mM 2-mercaptoethanol (Gibco), 100 U/mL penicillin, and 100 μg/mL streptomycin (Corning). From day 0 to day 3, ROCK inhibitor Y-27632 (Millipore) was added to the media at a final concentration of 20 μM. From day 0 to day 10, Wnt inhibitor IWR1 (Calbiochem) and TGFβ inhibitor SB431542 (Stem Cell Technologies) were added at a concentration of 3 μM and 5 μM, respectively. From day 10, the floating aggregates were cultured in ultra-low attachment culture dishes (Corning) under orbital agitation (50 rpm) in CDM II, containing DMEM/F12 medium (Gibco), 2mM Glutamax (Gibco), 1% N2 (Gibco), 1% Chemically Defined Lipid Concentrate (Gibco), 0.25 μg/mL fungizone (Gibco), 100 U/mL penicillin, and 100 μg/mL streptomycin. On day 18 cell aggregates were transferred to CDM III, consisting of CDM II supplemented with 10% fetal bovine serum (FBS) (GE-Healthcare), 5 μg/mL heparin (Sigma), and 1% Matrigel (Corning) and maintained at 50 rpm. From day 24 until day 50, organoids were cultured in CDM IV, consisting of CDM III supplemented with B27 supplement (Gibco) and 2% Matrigel.

### Confocal microscopy

To prepare organoids for immunohistochemistry, they were drop-fixed in 4% paraformaldehyde, dehydrated in 30% sucrose and embedded in Tissue-Tek using OCT compound (CAT#: 4583) and biopsy molds. Organoids were then serially cryosectioned onto slides at 30 μm. Thus, each slide from this sectioning contained 3–4 unique sections/Fields of View (FOV) per each organoid studied. Using this approach, we were able to robustly assess both independent and focal cell populations in each biological and technical organoid replicate. In further preparation, all sections underwent heat-mediated antigen retrieval in the citrate buffer and primary antibodies were incubated for each section overnight. Primary antibodies used were SOX2 (1:500, Abcam, ab92494), Beta-III-tubulin (1:500, Abcam, ab78078), Ki67 (1:250, ThermoFisher, CAT#: MA5–14520), PHC3 (1:200, ThermoFisher, CAT#: H00080012-B01P). Secondary antibodies were incubated for 2 h at room temperature and comprised antibodies for rabbit (Fluor 488 CAT#: A11008 & Fluor 633 CAT#: A21070), mouse (Fluor 488 CAT#: A11001 & Fluor 633 CAT#: A21052) and were used at a 1:2000 dilution and sourced from Life Technologies. Microscopy was completed on an Olympus IX81 Laser-Scanning Confocal Microscope, controlled by proprietary Olympus FluoView software. Images were typically acquired at 1200 × 1200-pixel resolution with optical *Z* slices (step sizes) ranging from 1 to 10 μm depending on the unit of analysis. All 16 lines were used for immunofluorescence quantifications.

### EV isolation

EVs were isolated from organoid media at 60 DIV. 10 days prior to media collection, the organoid media was changed to FBS-depleted CDM IV. CDM IV media was ultra-centrifuged at 100,000g overnight to remove FBS-derived vesicles. This ensures that the media collected at 60 DIV contains EVs derived solely from organoids. Cells were maintained in FBS-depleted CDM IV and at day 60 media was collected and processed using the Total Exosome Isolation Reagent (ThermoFisher CAT#: 4478359). Briefly, 10 mL of media was mixed with 5 mL of total exosome reagent and incubated at 4C overnight. The next day, samples were centrifuged at 10,000g for 1 hour and the pellet was resuspended in 1x PBS or RIPA buffer, depending on the downstream analysis. Samples resuspended in 1X PBS were used for RNA sequencing, TEM analysis and NTA while samples resuspended in RIPA buffer were used for proteomics and Western blot.

### Western blot

For Western blot, EVs were lysed using RIPA buffer and sonication. 30 μg of total protein of each sample was separated on 10% SDS-PAGE under non-reducing conditions and transferred onto a nitrocellulose membrane (BioRad CAT#: 1620113). The membranes were blocked overnight with 5% milk at 4°C and incubated with primary antibodies (ABs) against CD63 (1:500, ThermoFisher, CAT#: 10628D) and CD9 (1:500, ThermoFisher, CAT#: MA5–31980) for 2 h at RT. After washing, the blots were incubated with HRP-conjugated anti-mouse and anti-rabbit IgG secondary antibody (1:2000, IRDye^®^ 680RD, CAT#: 926–68070 & IRDye^®^ 800CW CAT#: 926–32210, Licor) for 1 h at RT. Protein expression was visualized using the Licor Odyssey^®^ M Imaging system.

### Transmission electron microscopy imaging

For negative staining TEM analysis, 5 μL of EVs in PBS (0.1 μg/μL) were placed on a glow-discharge formvar and carbon-coated 400 mesh grid and allowed to settle for 1 min. The sample was blotted and negatively stained with 50 μL of aqueous uranyl acetate (1.5%). After 1 min, the grid was blotted and air-dried for 5 min. Grids were imaged using a JEM-1400 transmission electron microscope (JEOL, USA, Inc., Peabody, MA) operated at 100 kV and images were captured on a Veleta 2K × 2K CCD camera (EM-SIS, Germany).

### Nanoparticle tracking analysis

NTA was performed using a previously established protocol (111, 112). All samples were diluted in PBS to a final volume of 1 ml. Ideal measurement concentrations were found by pre-testing the ideal particle per frame value (20–100 particles/frame). Following settings were set according to the manufacturer’s software manual (NanoSight NS500 User Manual, MAN0513–06-EN-00, 2016): camera level was increased until all particles were distinctly visible not exceeding a particle signal saturation over 20%, camera level 11, BLUE405, 1498 frames. Autofocus was adjusted so that indistinct particles were avoided. For each measurement, five 1-min videos were captured under the following conditions: cell temperature: 24°C; Syringe speed: 40 μl/s. After capture, the videos have been analyzed by the in-build NanoSight Software NTA 3.4 Build 3.4.003 with a detection threshold 5–18.

### Small RNA library preparation

Small RNA libraries were generated using NEXTFlex Small RNA Library Prep Kit v4 (Revvity). This kit is designed to capture short RNAs (17–65 nucleotides) with 5’ monophosphorylated and 3’ hydroxylated ends. The kit also allows for the capture of larger molecules (<200 bp) by using the no size selection protocol. The manufacturer’s instructions were followed through PCR amplification including 3’ adenylated adapter ligation, 5’ adapter ligation and RT first strand synthesis. Following PCR amplification, the libraries were cleaned up using the provided magnetic beads and the resulting pellet was resuspended in 11 μL of ultra-pure water. Sample concentrations were quantified using a NanoDrop Spectrophotometer, QC was evaluated using an Agilent Bioanalyzer Sample and ran on a picogel.

### RNA sequencing

The small RNAs from exosomes were isolated using the Total Exosome RNA & Protein Isolation Kit (ThermoFisher). Purified small RNA integrity was checked using a small RNA QC kit on 2100 Bioanalyzer (Agilent Technologies, Santa Clara, CA), and the concentration was measured using the Qubit Fluorometer (ThermoFisher Scientific, Inc., CA). Preparation of small RNA sample library and sequencing were performed by the Genomics Resources Core Facility at Weill Cornell Medicine using the NEXTFLEX^®^ Small RNA-Seq Kit v4 with UDIs kit (Revvity Inc.,), according to the manufacturer’s instructions. The normalized cDNA libraries were pooled and sequenced on Illumina NovaSeq X Plus sequencer with pair-end 50 cycles. The raw sequencing reads in BCL format were processed through bcl2fastq 2.19 (Illumina) for FASTQ conversion and demultiplexing.

### Bioinformatics pipeline for RNA sequencing

Approximately 5–20 million paired-end reads were generated per sample. Sequencing adapters and duplicate sequence reads were removed, and low-quality reads trimmed using *Trimmomatic* (version 0.39). Cleaned reads were aligned to the reference human genome (version GRCh38.99) using *STAR* alignment (version 2.7.10b). Read counts of genetic features were obtained by the *SubRead* package (version 2.0.3). The *DESeq2* R/Bioconductor package (version 1.44.0) was used to normalize count data and identify the differentially expressed genes (DEGs) between control and ASD samples. Significant differentially expressed genes were defined by a p value <0.05 and a log_2_ fold change >1.5 as thresholds. A minimum threshold of expression was set at 25%, representing the number of samples with non-zero expression values that would be considered for differential analysis. Employing these parameters, 177 differentially expressed genes and RNA elements were identified. Gene Ontology and pathway analyses were performed using the *clusterProfiler* package (version 4.12.6) in R. Cutoffs of significance were based on p-value and Benjamini-Hochberg (BH) adjusted p-value. All statistical analyses were performed in R (version 4.4.1). Figures were generated using *ggplot2* (version 3.5.1), *DESeq2* (version 1.44.0), *EnhancedVolcano* (version 1.22.0), and *enrichplot* (version 1.24.2).

### Sample preparation and label free proteomics

The protein samples were acetone precipitated and re-suspended in 0.1% RapiGest (Waters), 25 mM ammonium bicarbonate. The samples were then reduced with DTT, alkylated with iodoacetamide, and digested overnight with trypsin at 37 °C. The digests were desalted by C18 Stage-tip columns. The digests were analyzed using a Thermo Fisher Scientific EASY-nLC 1200 coupled on-line to a Fusion Lumos mass spectrometer (ThermoFisher Scientific). Buffer A (0.1% FA in water) and buffer B (0.1% FA in 80 % ACN) were used as mobile phases for gradient separation. A 75 μm × 15 cm chromatography column (ReproSil-Pur C18-AQ, 3 μm, Dr. Maisch GmbH, Germany) was packed in-house for peptide separation. Peptides were separated with a gradient of 5–40% buffer B over 30 min, 40–100% B over 10 min at a flow rate of 400 nL/min. The Fusion Lumos mass spectrometer was operated in a data independent acquisition (DIA) mode. MS1 scans were collected in the Orbitrap mass analyzer from 350–1400 m/z at 120K resolutions. The instrument was set to select precursors in 45 × 14 m/z wide windows with 1 m/z overlap from 350–975 m/z for HCD fragmentation. The MS/MS scans were collected in the orbitrap at 15K resolution. Data were searched against the human Uniprot database (8/7/2021) using DIA-NN v1.8 and filtered for 1% false discovery rate for both protein and peptide identifications

### Bioinformatics pipeline for proteomics analysis

Perseus (v2.0.7.0) (PMID: 27348712) was applied for downstream data processing and analysis. Data quality thresholds included protein group quantification at a 40% inclusion rate in at least one group (n=8). Protein intensities were log2 transformed and normalized through quantile normalization, with missing values imputed from normal distribution (width 0.3, downshift 1.8). Proteins were subjected to PCA and hierarchical clustering using an unpaired student’s t-test using Euclidean distance and average linkage clustering (*p* <0.05). Proteins were Z-score normalized. Gene Ontology (GO) functional enrichment and network/pathway analysis were conducted using gProfiler and enrichR databases for GO biological processes, cellular components, molecular functions, and KEGG pathways. Enrichment analysis was restricted to the top 10 categories with the highest statistically significant odds ratios comparing ASD and control groups (p < 0.05; PMID: 31066453, PMID: 27141961). PCA plots and heatmaps were generated in Perseus. Bar and violin plots were created in GraphPad Prism (v10.0.2) or Microsoft Excel. Comparative proteome analysis was conducted using the Simons Foundation Autism Research Initiative (SFARI) online database (PMID: 24090431).

## Supplementary Files

This is a list of supplementary files associated with this preprint. Click to download.

- 177DEGdataupdated.xlsx
- allsignificantmiRNAs.xlsx
- 362differentiallyregulatedproteins.xlsx
- SupplementaryMaterial.pdf

## Data Availability

RNA sequencing and proteomics data were deposited in dbGaP and PRIDE (only proteomics).
